# Supplementary material for: Nuclear myosin 1 activates p21 gene transcription in response to DNA damage through a chromatin-based mechanism
Source: Commun Biol. 2020 Mar 11;3:115. doi: 10.1038/s42003-020-0836-1 (PMC7066169; doi:10.1038/s42003-020-0836-1)
Supplement: Supplementary file 1 — Supplementary Information [file 42003_2020_836_MOESM1_ESM.pdf]

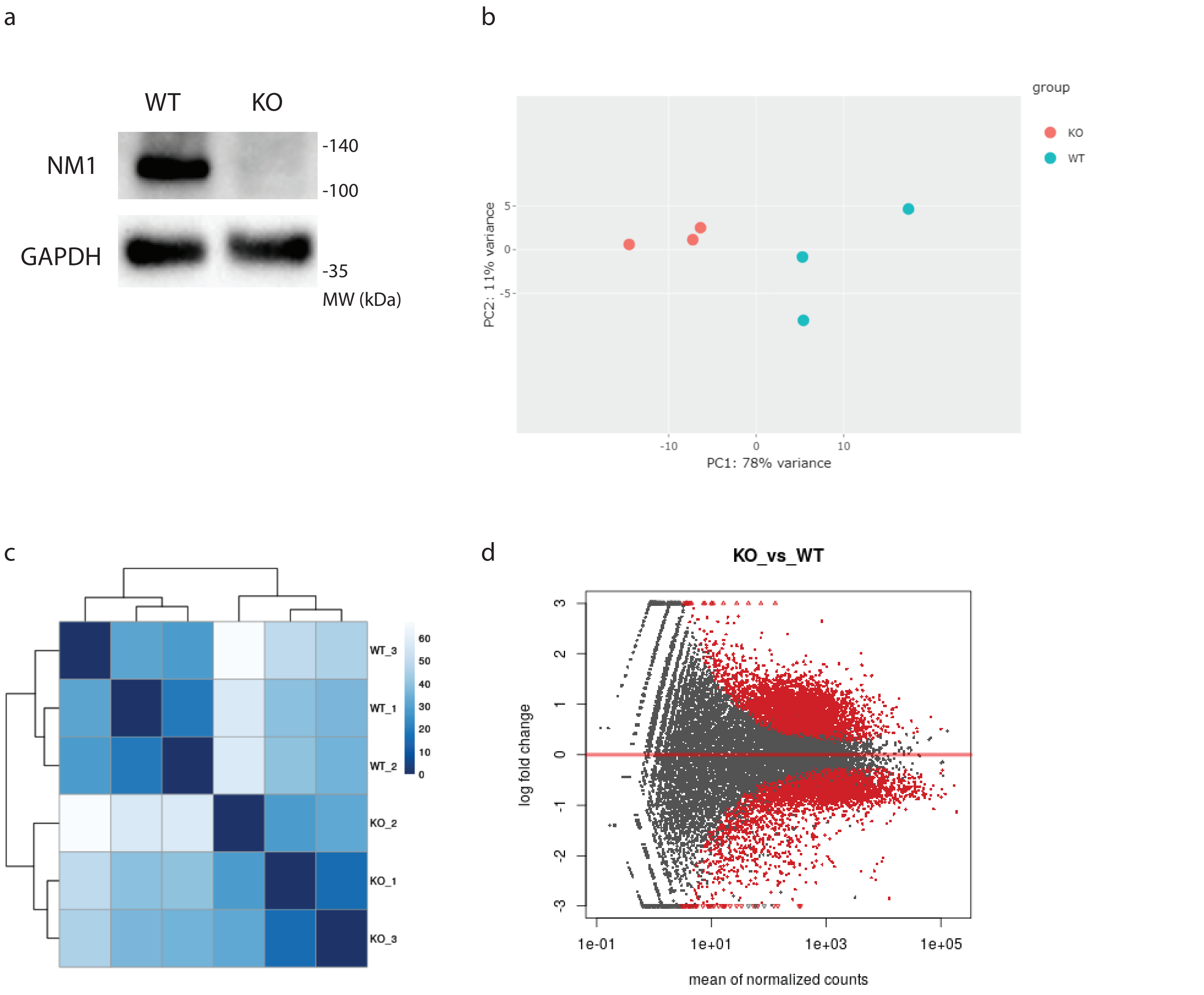

Supplementary Fig. 1. (a) Western blot analysis of primary mouse embryonic fibroblast derived from WT and NM1 KO embryos. (b) Principal component analysis (PCA) plots and distance heatmap (c) show that WT and KO samples cluster apart indicating higher variability between experimental groups than between samples within the same groups. (d) The differences in gene expression between WT and KO cells were visualized by MA plot by plotting the log2 (fold change between KO and WT condition) versus the mean of normalized counts. Genes showed in grey do not show any significant change in their expression, while genes marked red are differentially expressed.

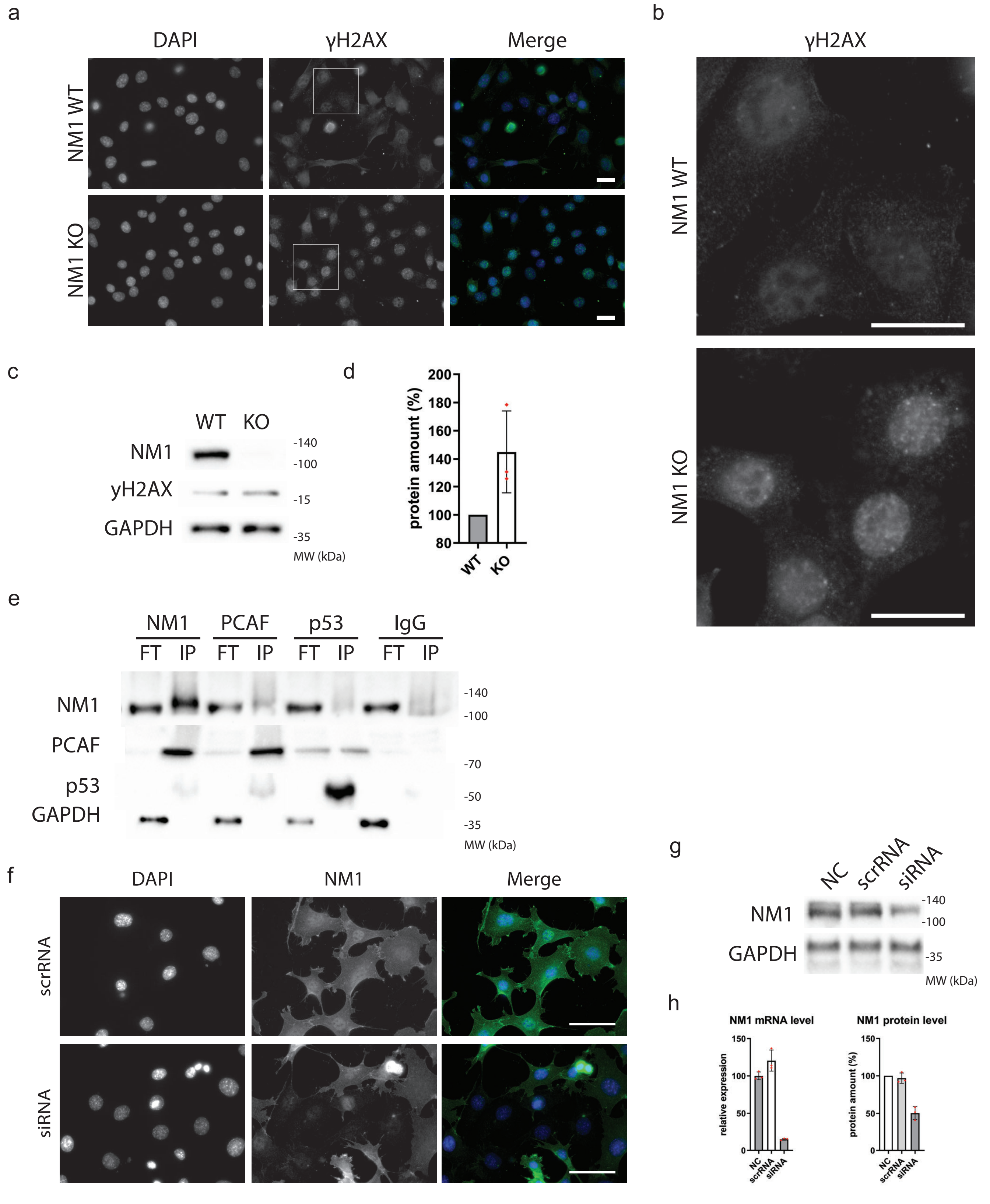

Supplementary Fig. 2. (a) Immunofluorescent staining of NM1 WT and KO cMEFs with an anti-γH2AX antibody. Nuclei are visualized by DAPI staining. Squares represents areas enlarged in (b). Scale bar is 5µm in both cases. (c) Western blot analysis of WT and KO cMEFs using anti-NM1, anti-γH2AX and anti-GAPDH antibodies. (d) Quantification of γH2AX protein level in WT and KO cMEFs from western blots. n=3 (e) Co-immunoprecipitation of proteins from untreated cells with antibodies against NM1, PCAF, p53 or control rabbit IgG and subsequent western blot analysis. (f) Immunofluorescent staining with DAPI and anti-NM1 antibodies of NM1 WT (scrRNA) and knockdown (KD) MEFs (siRNA). Scale bar is 10µm (g) Western blot analysis of WT and KD MEFs using anti-NM1 and anti-GAPDH antibodies. (h) Quantification of NM1 mRNA expression by qPCR relative to GAPDH expression and quantification of NM1 protein level by western blot. n=3

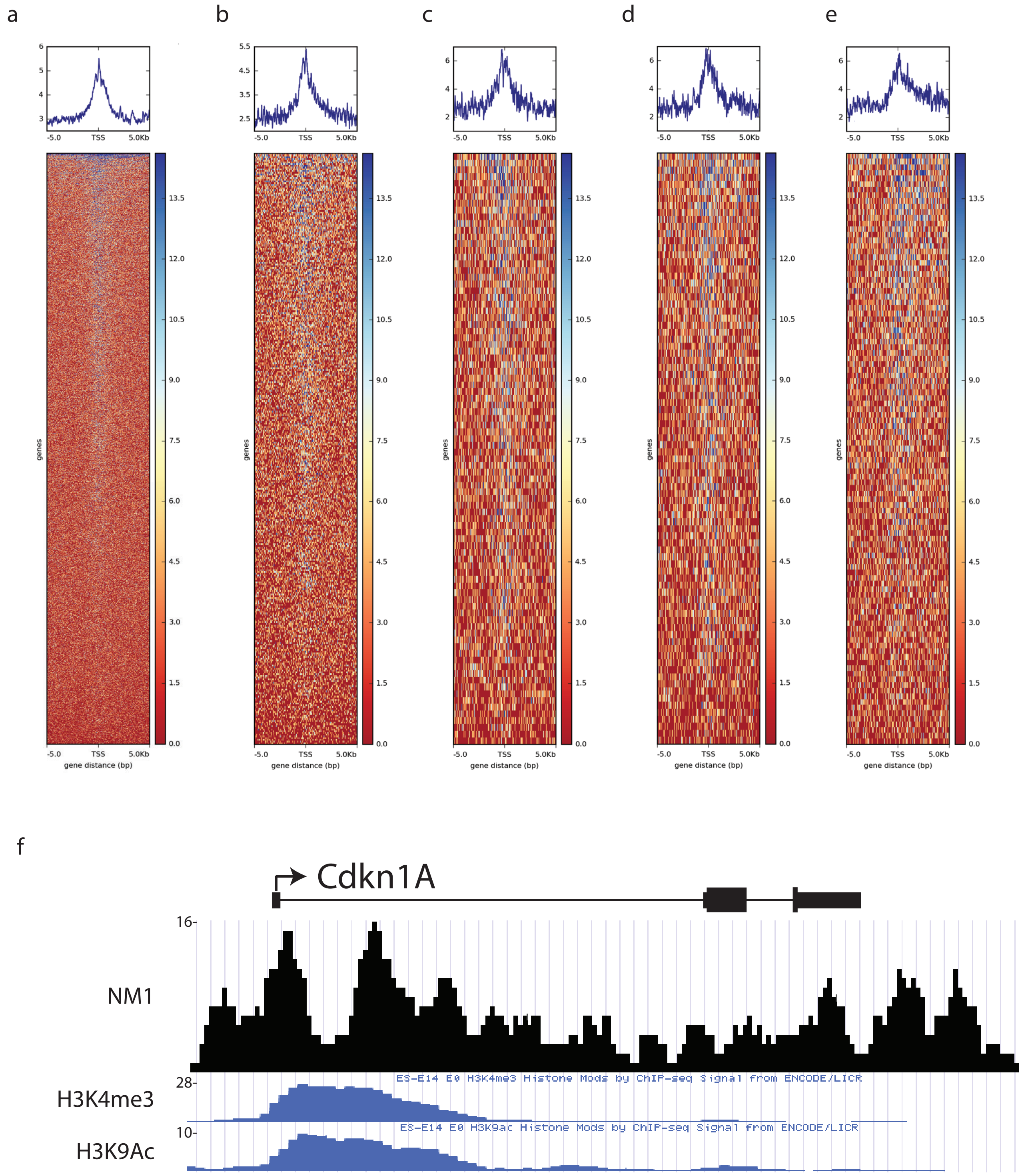

Supplementary Fig. 3. NM1 occupancy around transcription start sites (TSS) of selected gene groups analyzed by ChIP-Seq using an anti-NM1 antibody. (a) Density map of ChIP-seq reads for NM1 ±5 kb of the TSSs of all up- and down-regulated genes by at least 2-fold in KO cells. Scale bar, normalized RPKM. Top: shows the mean NM1 signal ±5 kb around the TSSs. The same analysis was performed on differentially expressed genes by at least 2-fold in KO cells belonging to gene ontology groups "Regulation of transcription, DNA-templated" (b), "cell cycle" (c) and "DNA damage" (d) or on p53-regulated genes (e). (f) NM1 occupancy profile across the p21 gene as revealed by ChIP-Seq correlates with H3K4me3 and H3K9ac occupancies. The y-axis represents RPKM per bin. Gene position (exon: box, intron: line) are shown above the tracks.

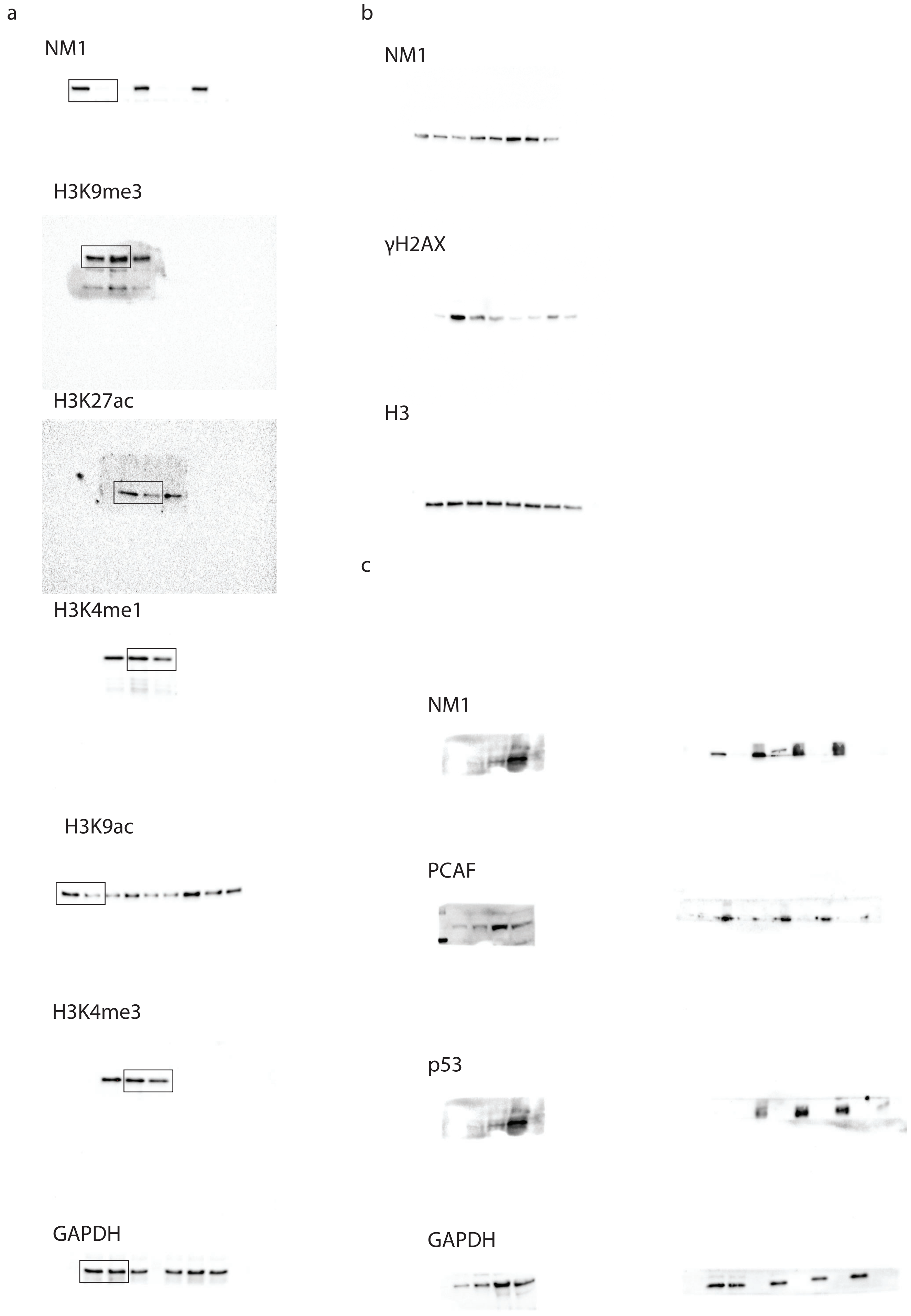

Supplementary Fig. 4. Full blots of raw data for Western analysis in Fig. 1c (a), Fig. 3e (b) and Fig. 5a (c)

Supplementary Table 1. Full list of gene ontology terms under "Biological process" group. All differentially expressed genes with at least 2-fold change in their expression were used for the gene ontology analysis.

Biology process Gene Ontology Term	Gene Count	Adj p-value	-LOG(p-value)
GO:0006355~regulation of transcription, DNA-templated	342	7.25E-19	18.13942219
GO:0003009~skeletal muscle contraction	17	1.16E-07	6.935577828
GO:0007049~cell cycle	105	6.24E-07	6.204759438
GO:0006351~transcription, DNA-templated	245	5.69E-06	5.244868655
GO:0006281~DNA repair	63	7.22E-06	5.141478888
GO:0006974~cellular response to DNA damage stimulus	76	9.35E-06	5.029038409
GO:0051301~cell division	68	4.25E-05	4.371920053
GO:0045893~positive regulation of transcription, DNA-templated	90	3.27E-04	3.484850243
GO:0045944~positive regulation of transcription from RNA polymerase II promoter	137	5.08E-04	3.294129448
GO:0007067~mitotic nuclear division	50	0.0024507	2.610709819
GO:0043524~negative regulation of neuron apoptotic process	34	0.002952856	2.529757692
GO:0007507~heart development	47	0.004329423	2.363569987
GO:0008380~RNA splicing	43	0.012413529	1.906104741
GO:0007519~skeletal muscle tissue development	17	0.014639587	1.83447119
GO:0045214~sarcomere organization	12	0.017161789	1.765437431
GO:0060048~cardiac muscle contraction	15	0.019484788	1.710304305
GO:0051298~centrosome duplication	10	0.020051923	1.697843982
GO:0019886~antigen processing and presentation of exogenous peptide antigen via MHC class II	8	0.026571825	1.575578617
GO:0006937~regulation of muscle contraction	10	0.026995583	1.568707281

Supplementary Table 2. Distribution of genes with at least 2-fold change in their expression in NM1 KO cells within GO terms “Cell cycle”, “Cell division”, “DNA repair” and “Cell response to DNA damage stimulus”.

**GO term categories**
**(a) Cell cycle**
**(b) Cell division**
**(c) DNA repair**
**(d) Cell response to DNA damage stimulus**

GO term	ENSEMBL Gene ID	Gene symbol	Log2(FC)	Adj p-value
(a)	ENSMUSG00000022034	Esco2	1.71125769	1.75E-07
(a)	ENSMUSG00000028282	Casp8ap2	1.551348034	3.25E-11
(a)	ENSMUSG00000024235	Map3k8	1.387446697	0.01929246
(a)	ENSMUSG00000027959	Sass6	1.376733124	0.000280099
(a)	ENSMUSG00000037225	Fgf2	1.367279237	0.039146925
(a)	ENSMUSG00000027641	Rbl1	1.31450547	3.49E-08
(a)	ENSMUSG00000031314	Taf1	1.248755035	2.18E-09
(a)	ENSMUSG00000040760	Appl1	1.242777686	8.76E-08
(a)	ENSMUSG00000020516	Rps6kb1	1.230871994	1.03E-07
(a)	ENSMUSG00000033054	Npat	1.226229247	3.94E-08
(a)	ENSMUSG00000042508	Dmtf1	1.202651622	1.98E-07
(a)	ENSMUSG00000021514	Zfp369	1.198549742	0.000306578
(a)	ENSMUSG00000030498	Gas2	1.193578183	6.68E-06
(a)	ENSMUSG00000029004	Kmt2e	1.192193869	1.83E-07
(a)	ENSMUSG00000022422	Dscc1	1.165604842	0.021949814
(a)	ENSMUSG00000027684	Mecom	1.157018372	4.58E-08
(a)	ENSMUSG00000020074	Ccar1	1.135993328	1.75E-07
(a)	ENSMUSG00000022369	Mtbp	1.132494303	2.05E-06
(a)	ENSMUSG00000003031	Cdkn1b	1.113385741	2.92E-07
(a)	ENSMUSG00000038344	Txlng	1.10901253	0.000385779
(a)	ENSMUSG00000037725	Ckap2	1.103049665	5.73E-12
(a)	ENSMUSG00000028693	Nasp	1.100089109	8.71E-10
(a)	ENSMUSG00000021134	Srsf5	1.064661573	1.01E-06
(a)	ENSMUSG00000031309	Rps6ka3	1.060224915	2.38E-06
(a)	ENSMUSG00000035455	Fignl1	1.048817799	4.02E-05
(a)	ENSMUSG00000040943	Tet2	1.041411013	0.008612205
(a)	ENSMUSG00000025907	Rb1cc1	1.034686623	1.18E-06
(a)	ENSMUSG00000030061	Uba3	1.032636754	1.02E-06
(a)	ENSMUSG00000002297	Dbf4	1.027695204	1.13E-12
(a)	ENSMUSG00000037664	Cdkn1c	-1.09937341	0.000932629
(a)	ENSMUSG00000018217	Pmp22	-1.09944598	1.36E-15
(a)	ENSMUSG00000029161	Cgref1	-1.27336672	1.64E-05
(a)	ENSMUSG00000022510	Trp63	-1.32067766	0.000619776
(a)	ENSMUSG00000031906	Smpd3	-1.81346517	1.58E-06
(a)	ENSMUSG00000026459	Myog	-2.09776303	0.025198841
(a)	ENSMUSG00000032968	Inha	-2.17285767	1.11E-07

(b)	ENSMUSG00000041498	Kif14	1.407968596	1.56E-10
(b)	ENSMUSG00000064128	Cenpj	1.306488498	1.61E-05
(b)	ENSMUSG00000020687	Cdc27	1.107296894	2.15E-09
(b)	ENSMUSG00000092074	Dynlt1a	-1.03671742	0.005114056
(c)	ENSMUSG00000029191	Rfc1	1.083442265	1.88E-08
(c)	ENSMUSG00000057113	Npm1	1.032903963	3.79E-12
(c)	ENSMUSG00000040455	Usp45	1.032024083	2.82E-06
(c)	ENSMUSG00000025612	Bach1	1.021893011	1.48E-05
(c)	ENSMUSG00000036611	Eepd1	-1.00505915	0.025738484
(d)	ENSMUSG00000017550	Atad5	1.479674207	1.50E-08
(d)	ENSMUSG00000037474	Dtl	1.402979577	4.09E-09
(d)	ENSMUSG00000020914	Top2a	1.399778224	3.49E-26
(d)	ENSMUSG00000000838	Fmr1	1.203460151	6.48E-09
(d)	ENSMUSG00000078671	Chd2	1.204433661	4.59E-06
(d)	ENSMUSG00000078773	Rad54b	1.122985626	0.014339615
(d)	ENSMUSG00000025899	Alkbh8	1.103785558	0.000331767
(d)	ENSMUSG00000061175	Fnip2	1.076536669	4.43E-08
(d)	ENSMUSG00000029238	Clock	1.07711291	5.78E-06
(d)	ENSMUSG00000005871	Apc	1.066374352	1.36E-06
(d)	ENSMUSG00000031628	Casp3	1.00619731	3.55E-09
(d)	ENSMUSG00000036278	Macrod1	-1.02872404	0.002191828
(d)	ENSMUSG00000035105	Egln3	-1.0928509	0.000210195
(d)	ENSMUSG00000034266	Batf	-1.20463002	0.031876678
(a) (b)	ENSMUSG00000026039	Sgo2a	1.909563191	6.48E-07
(a) (b)	ENSMUSG00000033952	Aspm	1.775575754	4.25E-12
(a) (b)	ENSMUSG00000024795	Kif20b	1.634977084	3.91E-14
(a) (b)	ENSMUSG00000012443	Kif11	1.603421777	3.41E-17
(a) (b)	ENSMUSG00000028312	Smc2	1.573512833	2.45E-12
(a) (b)	ENSMUSG00000045328	Cenpe	1.572827733	8.49E-17
(a) (b)	ENSMUSG00000027326	Knl1	1.563390397	1.00E-07
(a) (b)	ENSMUSG00000047534	Mis18bp1	1.517024431	1.49E-08
(a) (b)	ENSMUSG00000042029	Ncapg2	1.509516693	7.57E-13
(a) (b)	ENSMUSG00000028212	Ccne2	1.507953072	6.79E-07
(a) (b)	ENSMUSG00000028549	Itgb3bp	1.48940185	0.031365882
(a) (b)	ENSMUSG00000079555	Haus3	1.480637897	5.00E-08
(a) (b)	ENSMUSG00000057110	Cntrl	1.468431833	3.10E-08
(a) (b)	ENSMUSG00000034349	Smc4	1.45192783	1.61E-16
(a) (b)	ENSMUSG00000025001	Hells	1.419697178	3.03E-10
(a) (b)	ENSMUSG00000027550	Lrrcc1	1.390931974	1.26E-08
(a) (b)	ENSMUSG00000027379	Bub1	1.384823294	3.65E-13
(a) (b)	ENSMUSG00000024989	Cep55	1.340374265	1.44E-09
(a) (b)	ENSMUSG00000029283	Cdc7	1.334398549	1.60E-06
(a) (b)	ENSMUSG00000069910	Spdl1	1.311022532	6.51E-09
(a) (b)	ENSMUSG00000066842	Hmcn1	1.272988648	6.95E-09
(a) (b)	ENSMUSG00000020492	Ska2	1.267818894	2.12E-08
(a) (b)	ENSMUSG00000026088	Mitd1	1.265919516	1.59E-05
(a) (b)	ENSMUSG00000026349	Ccnt2	1.265146821	1.58E-07
(a) (b)	ENSMUSG00000025925	Terf1	1.256138736	7.08E-05
(a) (b)	ENSMUSG00000026683	Nuf2	1.255075727	1.48E-10

(a) (b)	ENSMUSG00000019773	Fbxo5	1.253415838	3.40E-09
(a) (b)	ENSMUSG00000024056	Ndc80	1.232028251	1.64E-07
(a) (b)	ENSMUSG00000027306	Nusap1	1.2301627	3.44E-07
(a) (b)	ENSMUSG00000006005	Tpr	1.214905394	2.19E-08
(a) (b)	ENSMUSG00000021537	Cetn3	1.207782895	4.45E-07
(a) (b)	ENSMUSG00000031010	Usp9x	1.196779331	9.50E-08
(a) (b)	ENSMUSG00000027699	Ect2	1.19635231	1.25E-12
(a) (b)	ENSMUSG00000023940	Sgo1	1.193653756	6.44E-08
(a) (b)	ENSMUSG00000029253	Cenpc1	1.186376171	5.65E-09
(a) (b)	ENSMUSG00000040549	Ckap5	1.179993853	1.50E-11
(a) (b)	ENSMUSG00000011831	Evi5	1.143930743	6.97E-07
(a) (b)	ENSMUSG00000041408	Wapl	1.124679756	3.45E-08
(a) (b)	ENSMUSG00000071350	Setdb2	1.119074357	0.000879931
(a) (b)	ENSMUSG00000024073	Birc6	1.114930938	3.03E-08
(a) (b)	ENSMUSG00000022978	Mis18a	1.112562071	4.61E-05
(a) (b)	ENSMUSG00000026276	Sept2	1.099680125	1.88E-08
(a) (b)	ENSMUSG00000033392	Clasp2	1.089021917	1.53E-06
(a) (b)	ENSMUSG00000032254	Kif23	1.080402023	1.31E-13
(a) (b)	ENSMUSG00000033502	Cdc14a	1.061962646	0.004605975
(a) (b)	ENSMUSG00000036777	Anln	1.055690048	1.11E-17
(a) (b)	ENSMUSG00000022070	Bora	1.050087626	2.43E-05
(a) (b)	ENSMUSG00000061665	Cd2ap	1.040444725	1.24E-05
(a) (b)	ENSMUSG00000045038	Prkce	1.028061461	0.000470685
(a) (b)	ENSMUSG00000032400	Zwilch	1.026745785	2.71E-06
(a) (b)	ENSMUSG00000001833	Sept7	1.023457115	9.09E-12
(a) (b)	ENSMUSG00000034165	Ccnd3	-1.00945581	0.000118684
(a) (b)	ENSMUSG00000020486	Sept4	-1.02226256	0.002360257
(a) (b)	ENSMUSG00000020782	Llgl2	-1.44386307	2.38E-11
(b) (c)	ENSMUSG00000029202	Pds5a*	1.201910032	1.39E-09
(c) (d)	ENSMUSG00000044702	Palb2	1.863631886	1.57E-05
(c) (d)	ENSMUSG00000034206	Polq	1.445801595	3.40E-07
(c) (d)	ENSMUSG00000031004	Mki67	1.79933243	2.33E-17
(c) (d)	ENSMUSG00000051235	Gen1	1.519385037	6.16E-06
(c) (d)	ENSMUSG00000019841	Rev3l	1.426095931	9.92E-08
(c) (d)	ENSMUSG00000022672	Prkdc	1.22003098	0.000133917
(c) (d)	ENSMUSG00000035365	Parpbp	1.39941138	3.46E-06
(c) (d)	ENSMUSG00000090112	Shprh	1.353053025	2.20E-09
(c) (d)	ENSMUSG00000032409	Atr	1.291894951	3.45E-07
(c) (d)	ENSMUSG00000043535	Setx	1.373700818	4.14E-10
(c) (d)	ENSMUSG00000047757	Fancb	1.315244506	0.000211638
(c) (d)	ENSMUSG00000039396	Neil3	1.633939602	0.000703607
(c) (d)	ENSMUSG00000030346	Rad51ap1	1.290324138	9.33E-06
(c) (d)	ENSMUSG00000031229	Atrx	1.41187585	5.51E-07
(c) (d)	ENSMUSG00000030655	Smg1	1.242009981	4.22E-10
(c) (d)	ENSMUSG00000030528	Blm	1.273197151	3.56E-07
(c) (d)	ENSMUSG00000040865	Ino80d	1.347529884	7.56E-09
(c) (d)	ENSMUSG00000020608	Smc6	1.247215661	1.78E-09
(c) (d)	ENSMUSG00000035726	Supt16	1.306438017	1.31E-10
(c) (d)	ENSMUSG00000028560	Usp1	1.183301369	1.30E-14

(c) (d)	ENSMUSG00000061755	Bod1l	1.172778402	1.95E-08
(c) (d)	ENSMUSG00000055884	Fancm	1.186915058	3.31E-07
(c) (d)	ENSMUSG00000017291	Taok1	1.130463947	6.54E-07
(c) (d)	ENSMUSG00000069495	Epc2	1.19314929	2.27E-07
(c) (d)	ENSMUSG00000026196	Bard1	1.092585576	0.001476677
(c) (d)	ENSMUSG00000040204	Pclaf	1.125698623	9.31E-15
(c) (d)	ENSMUSG00000054051	Ercc6	1.152473285	1.23E-05
(c) (d)	ENSMUSG00000039748	Exo1	1.142964897	1.97E-07
(c) (d)	ENSMUSG00000037355	Uvssa	1.133401711	1.34E-05
(c) (d)	ENSMUSG00000032555	Topbp1	1.092956409	4.32E-12
(c) (d)	ENSMUSG00000038774	Ascc3	1.096053881	8.51E-06
(c) (d)	ENSMUSG00000012483	Rpa3	1.091175918	5.60E-05
(c) (d)	ENSMUSG00000026082	Rev1	1.090772336	4.55E-08
(c) (d)	ENSMUSG00000030254	Rad18	1.078147681	2.37E-08
(c) (d)	ENSMUSG00000035401	Emsy	1.07744376	7.91E-08
(c) (d)	ENSMUSG00000026648	Dclre1c	1.072730394	0.001601497
(c) (d)	ENSMUSG00000021597	Ankrd32	1.059630232	0.002238719
(c) (d)	ENSMUSG00000030322	Mbd4	1.035245167	0.004381754
(c) (d)	ENSMUSG00000034329	Brip1	1.039915227	0.001129563
(c) (d)	ENSMUSG00000025878	Uimc1	1.028796906	5.56E-05
(c) (d)	ENSMUSG00000035234	Abraxas1	1.022349089	0.008214339
(c) (d)	ENSMUSG00000021470	Ercc6l2	1.018837521	8.05E-06
(c) (d)	ENSMUSG00000017897	Eya2	-1.0658211	0.014777507
(c) (d)	ENSMUSG00000054612	Mgmt	-1.07027509	0.003450678
(a) (b) (c)	ENSMUSG00000034021	Pds5b*	1.298188038	2.46E-12
(a) (b) (d)	ENSMUSG00000031644	Nek1*	1.366192385	7.81E-05
(a) (b) (d)	ENSMUSG00000026779	Mastl*	1.220412317	1.91E-08
(a) (c) (d)	ENSMUSG00000032113	Chek1*	1.566094962	4.19E-07
(a) (c) (d)	ENSMUSG00000017146	Brca1*	1.472344273	1.31E-08
(a) (c) (d)	ENSMUSG00000034218	Atm*	1.357826304	9.45E-10
(a) (c) (d)	ENSMUSG00000028224	Nbn*	1.108696916	1.36E-07
(a) (c) (d)	ENSMUSG00000020380	Rad50*	1.061644738	6.87E-07
(a) (c) (d)	ENSMUSG00000042489	Clspn*	1.281453016	6.20E-09
(a) (c) (d)	ENSMUSG00000041147	Brca2*	1.290020428	0.000105344
(a) (b) (c) (d)	ENSMUSG00000024943	Smc5*	1.435523352	5.32E-07
(a) (b) (c) (d)	ENSMUSG00000024974	Smc3*	1.37505807	2.74E-11
(a) (b) (c) (d)	ENSMUSG00000041133	Smc1a*	1.282471991	2.76E-13
(a) (b) (c) (d)	ENSMUSG00000049717	Lig4*	1.117129153	8.05E-06
(a) (b) (c) (d)	ENSMUSG00000034154	Ino80*	1.098229249	1.76E-06
(a) (b) (c) (d)	ENSMUSG00000041238	Rbbp8*	1.082292963	6.77E-06

Supplementary Table 3. Differentially expressed genes in NM1 KO cells related to “Cell cycle”, “DNA repair” and “Cell response to DNA damage stimulus” GO terms which are

GO term categories
(a) Cell cycle
(b) DNA repair
(c) Cell response to DNA damage stimulus

GO term	ENSEMBL Gene ID	Gene symbol	Log2(FC)	Adj p-value
(a)	ENSMUSG00000000001	Gnai3	0.996941229	7.62E-08
(a)	ENSMUSG00000040021	Lats1	0.992505061	3.00E-06
(a)	ENSMUSG00000025862	Stag2	0.985716597	2.49E-07
(a)	ENSMUSG00000031878	Nae1	0.983639482	1.47E-08
(a)	ENSMUSG00000004591	Pkn2	0.979980767	1.48E-07
(a)	ENSMUSG00000014956	Ppp1cb	0.977841841	1.46E-10
(a)	ENSMUSG00000033364	Usp37	0.968367638	0.000151952
(a)	ENSMUSG00000021693	Kif2a	0.963140158	5.15E-10
(a)	ENSMUSG00000019256	Ahr	0.960854025	2.62E-05
(a)	ENSMUSG00000062510	Nsl1	0.941932089	0.000970428
(a)	ENSMUSG00000038943	Prc1	0.939045578	1.58E-12
(a)	ENSMUSG00000035310	Lin54	0.937098729	7.02E-06
(a)	ENSMUSG00000037286	Stag1	0.915517364	8.62E-06
(a)	ENSMUSG00000036779	Papd5	0.913333319	3.29E-05
(a)	ENSMUSG00000033900	Map9	0.906400923	0.000279853
(a)	ENSMUSG00000033713	Foxn3	0.902517222	0.001579369
(a)	ENSMUSG00000040599	Mis12	0.900196162	4.19E-07
(a)	ENSMUSG00000058729	Lin9	0.899715432	0.000215755
(a)	ENSMUSG00000026491	Ahctf1	0.896519017	8.07E-08
(a)	ENSMUSG00000031529	Tnks	0.891638119	3.78E-06
(a)	ENSMUSG00000029414	Kntc1	0.890939054	8.19E-07
(a)	ENSMUSG00000021965	Ska3	0.889671928	7.08E-05
(a)	ENSMUSG00000040860	Crocc	0.883959086	0.00010777
(a)	ENSMUSG00000042688	Mapk6	0.883064928	3.18E-05
(a)	ENSMUSG00000035024	Ncapd3	0.875202282	2.66E-06
(a)	ENSMUSG00000070923	Klhl9	0.874370162	5.18E-08
(a)	ENSMUSG00000026361	Cdc73	0.868872448	6.53E-05
(a)	ENSMUSG00000049327	Kmt5a	0.864936958	4.69E-07
(a)	ENSMUSG00000000708	Kat2b	0.863171531	0.001315128
(a)	ENSMUSG00000011960	Ccnt1	0.862489127	1.78E-06
(a)	ENSMUSG00000019988	Nedd1	0.861030494	1.50E-05
(a)	ENSMUSG00000032064	Dixdc1	0.8601254	0.000527328
(a)	ENSMUSG00000037544	Dlgap5	0.859497645	3.59E-06
(a)	ENSMUSG00000024817	Uhrf2	0.857840052	2.92E-07
(a)	ENSMUSG00000019917	Sept10	0.855627366	1.48E-05
(a)	ENSMUSG00000055024	Ep300	0.853455036	1.71E-07
(a)	ENSMUSG00000031242	2610002M06Rik	0.851318227	0.000422968
(a)	ENSMUSG00000004085	Map3k20	0.847026077	1.34E-06
(a)	ENSMUSG00000009907	Vps4b	0.843221519	1.43E-05
(a)	ENSMUSG00000022961	Son	0.840871546	5.18E-05
(a)	ENSMUSG00000028926	Cdk14	0.840015602	1.82E-07
(a)	ENSMUSG00000027339	Rassf2	0.837636714	0.026772284
(a)	ENSMUSG00000029516	Cit	0.835951871	4.06E-05
(a)	ENSMUSG00000031176	Dynlt3	0.825721071	0.000513279
(a)	ENSMUSG00000071533	Pcnp	0.825357383	8.63E-08
(a)	ENSMUSG00000045636	Mtus1	0.796443192	0.012430708
(a)	ENSMUSG00000029176	Anapc4	0.794112061	7.47E-08
(a)	ENSMUSG00000035437	Rabgap1	0.792940738	2.16E-06
(a)	ENSMUSG00000048922	Cdca2	0.787828557	1.68E-05
(a)	ENSMUSG00000027363	Usp8	0.770905267	2.18E-05
(a)	ENSMUSG00000052957	Gas1	0.765206306	1.00E-05
(a)	ENSMUSG00000022673	Mcm4	0.760939171	1.48E-08
(a)	ENSMUSG00000029684	Wasl	0.760114142	1.69E-05
(a)	ENSMUSG00000038822	Hace1	0.759679543	0.008202905
(a)	ENSMUSG00000024293	Esco1	0.755967885	0.020529321
(a)	ENSMUSG00000035351	Nup37	0.752526128	0.003004564
(a)	ENSMUSG00000026646	Suv39h2	0.749463316	0.031519836
(a)	ENSMUSG00000006715	Gmnn	0.746635058	0.001096575
(a)	ENSMUSG00000029910	Mad2l1	0.740339309	1.20E-05
(a)	ENSMUSG00000031666	Rbl2	0.731722344	0.001831831
(a)	ENSMUSG00000024576	Csnk1a1	0.730034039	1.81E-06
(a)	ENSMUSG00000046179	E2f8	0.729451798	0.002016351
(a)	ENSMUSG00000005233	Spc25	0.724869264	0.000343321
(a)	ENSMUSG00000005102	Eif2ak4	0.722856555	0.000318886
(a)	ENSMUSG00000040084	Bub1b	0.718403847	1.28E-06
(a)	ENSMUSG00000035469	Rcbtb1	0.717254493	9.31E-06
(a)	ENSMUSG00000075266	Cenpw	0.707203591	0.023192542
(a)	ENSMUSG00000028551	Cdkn2c	0.70193457	0.034982674
(a)	ENSMUSG00000031971	Ccsap	0.697707905	0.016869769
(a)	ENSMUSG00000040274	Cdk6	0.693307708	0.000128983
(a)	ENSMUSG00000021548	Ccnh	0.689344512	0.000370144
(a)	ENSMUSG00000051220	Ercc6l	0.685503352	0.000456719
(a)	ENSMUSG00000036977	Anapc10	0.682643055	0.021021237
(a)	ENSMUSG00000052062	Pard3b	0.677195833	0.0406557
(a)	ENSMUSG00000027285	Haus2	0.676432114	0.001545984

GO term	ENSEMBL Gene ID	Gene symbol	Log2(FC)	Adj p-value
(b)	ENSMUSG00000046295	Ankle1	0.982403767	0.016079119
(b)	ENSMUSG00000036023	Parp2	0.916404529	6.83E-05
(b)	ENSMUSG00000019857	Asf1a	0.886025085	8.46E-05
(b)	ENSMUSG00000030243	Recql	0.804753272	0.000161298
(b)	ENSMUSG00000027433	Xrn2	0.637377634	7.45E-06
(b)	ENSMUSG00000045482	Trrap	0.528301905	0.000191994
(b)	ENSMUSG00000058301	Upf1	0.518112542	0.000377879
(b)	ENSMUSG00000005566	Trim28	-0.508336707	0.010747865
(b)	ENSMUSG00000030689	Ino80e	-0.580955068	0.017484288
(b)	ENSMUSG00000018449	Rpain	-0.694953338	0.009733652
(b)	ENSMUSG00000010362	Rdm1	-0.916100844	0.009474129
(c)	ENSMUSG00000038069	Cdkn2aip	0.998821056	0.000331767
(c)	ENSMUSG00000033721	Vav3	0.997508095	0.002156107
(c)	ENSMUSG00000030779	Rbbp6	0.991554753	2.25E-06
(c)	ENSMUSG00000020063	Sirt1	0.972675002	6.50E-05
(c)	ENSMUSG00000021911	Parg	0.953491882	1.40E-06
(c)	ENSMUSG00000036822	Topors	0.943265508	1.59E-06
(c)	ENSMUSG00000027104	Atf2	0.941980341	6.46E-06
(c)	ENSMUSG00000002748	Baz1b	0.920770824	1.77E-09
(c)	ENSMUSG00000035764	Fbxo45	0.916170515	0.000123152
(c)	ENSMUSG00000027242	Wdr76	0.900502455	0.007716902
(c)	ENSMUSG00000020228	Helb	0.869411772	0.001421294
(c)	ENSMUSG00000024283	Wac	0.867344505	8.32E-07
(c)	ENSMUSG00000021245	Mlh3	0.859572462	0.017304516
(c)	ENSMUSG00000038369	Ncoa6	0.749981745	1.16E-05
(c)	ENSMUSG00000034342	Cbl	0.738525482	0.002549156
(c)	ENSMUSG00000061759	Armt1	0.713850153	0.000239336
(c)	ENSMUSG00000058761	Rnf169	0.711363024	0.000175541
(c)	ENSMUSG00000064289	Tank	0.702470812	0.000190801
(c)	ENSMUSG00000024521	Pmaip1	0.661633472	0.002714083
(c)	ENSMUSG00000040433	Zbtb38	0.628726542	0.011180967
(c)	ENSMUSG00000037111	Setd7	0.626799643	0.000218393
(c)	ENSMUSG00000038005	Hpf1	0.62658934	0.000813037
(c)	ENSMUSG00000020546	Stxbp4	0.622447458	0.006129364
(c)	ENSMUSG00000028086	Fbxw7	0.590674969	0.017887136
(c)	ENSMUSG00000027164	Traf6	0.51391341	0.013985774
(c)	ENSMUSG00000020898	Ctc1	-0.535910722	0.002275919
(c)	ENSMUSG00000001924	Uba1	-0.607278596	3.78E-05
(c)	ENSMUSG00000019969	Psen1	-0.645143162	4.81E-05
(c)	ENSMUSG00000024429	Gnl1	-0.702426178	0.000147022
(c)	ENSMUSG00000001729	Akt1	-0.729399647	3.43E-05
(c)	ENSMUSG00000002661	Alkbh7	-0.770501252	0.005064014
(c)	ENSMUSG00000037032	Apbb1	-0.770884308	0.002365413
(c)	ENSMUSG00000016528	Mapkapk2	-0.787622821	4.55E-06
(c)	ENSMUSG00000030086	Chchd6	-0.801534948	0.009583763
(c)	ENSMUSG00000003873	Bax	-0.841153491	0.002046812
(a) (b)	ENSMUSG00000000902	Smarcb1	-0.544164895	0.017342225
(a) (c)	ENSMUSG00000021918	Nek4	0.887565334	0.006131325
(a) (c)	ENSMUSG00000041997	Tlk1	0.869382937	8.72E-06
(a) (c)	ENSMUSG00000000355	Mcts1	0.853928055	0.000317955
(a) (c)	ENSMUSG00000032397	Tipin	0.849583359	1.25E-05
(a) (c)	ENSMUSG00000025616	Usp16	0.830152386	0.000240882
(a) (c)	ENSMUSG00000020694	Tlk2	0.801024671	1.59E-05
(a) (c)	ENSMUSG00000036390	Gadd45a	0.732346094	0.022450659
(a) (c)	ENSMUSG00000028680	Plk3	-0.617954601	0.001241811
(a) (c)	ENSMUSG00000029003	Mad2l2	-0.645537059	0.010826731
(a) (c)	ENSMUSG00000000552	Zfp385a	-0.672828603	0.007085932
(a) (c)	ENSMUSG00000010067	Rassf1	-0.755849728	0.000821134
(a) (c)	ENSMUSG00000063065	Mapk3	-0.819778973	0.000125395
(a) (c)	ENSMUSG00000030538	Cib1	-0.902056723	0.000105398
(a) (c)	ENSMUSG00000035390	Brsk1	-0.910318547	1.49E-05
(b) (c)	ENSMUSG00000022906	Parp9	0.951176633	0.018655675
(b) (c)	ENSMUSG00000040850	Psme4	0.950909964	1.95E-08
(b) (c)	ENSMUSG00000014850	Msh3	0.944249559	0.000120674
(b) (c)	ENSMUSG00000059263	Usp47	0.939980339	8.49E-08
(b) (c)	ENSMUSG00000036097	Slf2	0.935528498	4.09E-07
(b) (c)	ENSMUSG00000035958	Tdp2	0.894656511	0.000143195
(b) (c)	ENSMUSG00000025932	Eya1	0.894648821	0.000126869
(b) (c)	ENSMUSG00000029920	Smarcad1	0.894380464	4.06E-06
(b) (c)	ENSMUSG00000033454	Zbtb1	0.883326664	0.000222751
(b) (c)	ENSMUSG00000037487	Ubr5	0.874756558	3.48E-06
(b) (c)	ENSMUSG00000031583	Wrn	0.861205217	0.000999281
(b) (c)	ENSMUSG00000021668	Polk	0.856226135	1.13E-07
(b) (c)	ENSMUSG00000033458	Fan1	0.854315599	0.005452383
(b) (c)	ENSMUSG00000028329	Xpa	0.853171558	0.009768513

(a)	ENSMUSG00000030965	Abraxas2	0.674096459	0.000926759
(a)	ENSMUSG00000020745	Pafah1b1	0.66253446	0.000117487
(a)	ENSMUSG00000058013	Sept11	0.660582693	2.84E-06
(a)	ENSMUSG00000044783	Hjurp; A730008H	0.658041479	3.60E-05
(a)	ENSMUSG00000036840	Siah1a	0.657680953	0.013660303
(a)	ENSMUSG00000021395	Spin1	0.651307243	8.27E-05
(a)	ENSMUSG00000044201	Cdc25c	0.644866707	0.014907419
(a)	ENSMUSG00000031660	Brd7	0.636059688	5.96E-05
(a)	ENSMUSG00000027469	Tpx2	0.620605976	6.44E-07
(a)	ENSMUSG00000040102	Klhl42	0.597681867	0.002249213
(a)	ENSMUSG00000031016	Wee1	0.597337809	0.004310663
(a)	ENSMUSG00000032782	Cntrob	0.59299817	0.009223145
(a)	ENSMUSG00000027635	Dsn1	0.584347074	0.009490434
(a)	ENSMUSG00000038241	Cep250	0.584230814	0.001171653
(a)	ENSMUSG00000021115	Vrk1	0.583137623	0.001187377
(a)	ENSMUSG00000032411	Tfdp2	0.579275308	0.008736487
(a)	ENSMUSG00000026622	Nek2	0.574020162	0.00078224
(a)	ENSMUSG00000029062	Cdk11b	0.562987388	0.00157887
(a)	ENSMUSG00000079614	Seh1l	0.557761568	0.000476266
(a)	ENSMUSG00000016128	Stard13	0.548317087	0.005023153
(a)	ENSMUSG00000017421	Zfp207	0.540435018	1.67E-05
(a)	ENSMUSG00000022772	Senp5	0.534257651	0.005282818
(a)	ENSMUSG00000062248	Cks2	0.525562066	0.000716892
(a)	ENSMUSG00000065954	Tacc1	0.523267466	0.016846299
(a)	ENSMUSG00000048170	Mcmbp	0.513937537	0.00072363
(a)	ENSMUSG00000091337	Eid1	0.506031964	0.001903471
(a)	ENSMUSG00000008859	Rala	0.503600495	0.004299335
(a)	ENSMUSG00000066979	Bub3	-0.501634069	0.011959789
(a)	ENSMUSG00000020107	Anapc16	-0.510616156	0.003186685
(a)	ENSMUSG00000023572	Ccndbp1	-0.514678622	0.003154171
(a)	ENSMUSG00000005699	Pard6a	-0.517846802	0.039049402
(a)	ENSMUSG00000021866	Anxa11	-0.531957071	0.020315648
(a)	ENSMUSG00000062234	Gak	-0.54617391	0.00011479
(a)	ENSMUSG00000031913	Vps4a	-0.552456016	0.002103081
(a)	ENSMUSG00000022750	Klhl22	-0.557912574	1.42E-05
(a)	ENSMUSG00000031729	Ist1	-0.565437439	3.29E-05
(a)	ENSMUSG00000039660	Spout1	-0.568145468	0.018786373
(a)	ENSMUSG00000026965	Anapc2	-0.568183983	0.007282714
(a)	ENSMUSG00000060860	Ube2s	-0.568718511	0.025689312
(a)	ENSMUSG00000006728	Cdk4	-0.571553111	0.013891294
(a)	ENSMUSG00000026027	Stradb	-0.576688591	0.002119742
(a)	ENSMUSG00000029394	Cdk2ap1	-0.580844077	0.0041701
(a)	ENSMUSG00000022178	Ajuba	-0.593823139	0.002078189
(a)	ENSMUSG00000038393	Txnip	-0.594386963	9.72E-05
(a)	ENSMUSG00000044502	Bod1	-0.600406684	0.0029971
(a)	ENSMUSG00000036106	Prr5	-0.637974541	0.003194568
(a)	ENSMUSG00000033088	Triobp	-0.653629912	5.96E-05
(a)	ENSMUSG00000068580	Zfyve19	-0.656855323	0.010338853
(a)	ENSMUSG00000028969	Cdk5	-0.664415801	0.003147216
(a)	ENSMUSG00000030397	Mark4	-0.671741646	0.000733473
(a)	ENSMUSG00000025135	Anapc11	-0.681609659	0.001672937
(a)	ENSMUSG00000055128	Cgrrf1	-0.683235152	0.000248145
(a)	ENSMUSG00000039130	Zc3hc1	-0.684319218	0.010681326
(a)	ENSMUSG00000072214	Sept5	-0.690645324	0.000534009
(a)	ENSMUSG00000046691	Chtf8	-0.695042671	0.001065005
(a)	ENSMUSG00000049680	Urgcp	-0.699522049	0.001751225
(a)	ENSMUSG00000011114	Tbrg1	-0.714600072	0.000182082
(a)	ENSMUSG00000069631	Strada	-0.720243013	0.000236499
(a)	ENSMUSG00000029466	Anapc7	-0.736396189	0.000302365
(a)	ENSMUSG00000056596	Trnp1	-0.738628877	0.00028753
(a)	ENSMUSG00000022089	Bin3	-0.738864039	4.11E-05
(a)	ENSMUSG00000031787	Katnb1	-0.749646929	0.003048293
(a)	ENSMUSG00000032562	Gnai2	-0.751271355	5.76E-06
(a)	ENSMUSG00000030272	Camk1	-0.753865991	0.000441044
(a)	ENSMUSG00000024944	Arl2	-0.758003102	0.000964331
(a)	ENSMUSG00000035828	Pim3	-0.759845555	0.007273915
(a)	ENSMUSG00000018821	Avpi1	-0.776959671	0.003633736
(a)	ENSMUSG00000040385	Ppp1ca	-0.779563703	0.004777702
(a)	ENSMUSG00000020372	Rack1	-0.810834752	0.000816675
(a)	ENSMUSG00000020307	Cdc34	-0.812779932	0.000591326
(a)	ENSMUSG00000032171	Pin1	-0.816788836	0.006029688
(a)	ENSMUSG00000020232	Hmg20b	-0.826922615	1.65E-06
(a)	ENSMUSG00000015149	Sirt2	-0.827291836	6.48E-09
(a)	ENSMUSG00000033751	Gadd45gip1	-0.833735237	0.006574381
(a)	ENSMUSG00000029166	Mapre3	-0.861255055	2.56E-06
(a)	ENSMUSG00000028447	Dctn3	-0.880601186	0.000672658
(a)	ENSMUSG00000000743	Chmp1a	-0.883446747	4.36E-05
(a)	ENSMUSG00000011589	Fsd1	-0.891030398	0.001024942
(a)	ENSMUSG00000024190	Dusp1	-0.905149229	7.62E-05
(a)	ENSMUSG00000004864	Mapk13	-0.930610653	0.015101642
(a)	ENSMUSG00000027510	Rbm38	-0.932832193	8.99E-05
(a)	ENSMUSG00000024790	Sac3d1	-0.946702013	1.33E-05
(a)	ENSMUSG00000002635	Pdcd2l	-0.973210343	0.000216216

(b) (c)	ENSMUSG00000021287	Xrcc3	0.846829454	0.003200794
(b) (c)	ENSMUSG00000022292	Rrm2b	0.844734297	0.005539819
(b) (c)	ENSMUSG00000018189	Uchl5	0.843082475	3.17E-05
(b) (c)	ENSMUSG00000021639	Gtf2h2	0.840815984	0.000839459
(b) (c)	ENSMUSG00000030451	Herc2	0.833610619	3.27E-06
(b) (c)	ENSMUSG00000045751	Mms22l	0.833534308	0.00018003
(b) (c)	ENSMUSG00000026219	Trip12	0.829056809	3.64E-06
(b) (c)	ENSMUSG00000014074	Rnf168	0.819650037	0.00010986
(b) (c)	ENSMUSG00000033102	Cdc14b	0.806734751	5.16E-05
(b) (c)	ENSMUSG00000022710	Usp7	0.800798872	2.84E-06
(b) (c)	ENSMUSG00000005370	Msh6	0.782072264	2.49E-07
(b) (c)	ENSMUSG00000020413	Hus1	0.773101522	0.00050093
(b) (c)	ENSMUSG00000006599	Gtf2h1	0.771228245	2.31E-07
(b) (c)	ENSMUSG00000020156	Mum1	0.73492519	1.01E-05
(b) (c)	ENSMUSG00000028933	Xrcc2	0.700754596	0.02479446
(b) (c)	ENSMUSG00000028820	Sfpq	0.698015042	9.70E-08
(b) (c)	ENSMUSG00000021690	Jmy	0.683304148	0.002507387
(b) (c)	ENSMUSG00000026048	Ercc5	0.68150199	2.30E-05
(b) (c)	ENSMUSG00000037262	Kin	0.679703396	0.005274277
(b) (c)	ENSMUSG00000025261	Huwe1	0.676496827	0.000284822
(b) (c)	ENSMUSG00000026107	Nabp1	0.664984904	0.001325482
(b) (c)	ENSMUSG00000031422	Morf4l2	0.653691048	5.59E-05
(b) (c)	ENSMUSG00000026914	Psmd14	0.648542793	1.26E-06
(b) (c)	ENSMUSG00000041064	Pif1	0.642736857	0.007184349
(b) (c)	ENSMUSG00000031928	Mre11a	0.615796547	0.000142173
(b) (c)	ENSMUSG00000028702	Rad54l	0.610960867	0.016703794
(b) (c)	ENSMUSG00000023953	Polh	0.599812741	0.003221009
(b) (c)	ENSMUSG00000020390	Ube2b	0.545006906	0.001205153
(b) (c)	ENSMUSG00000032815	Fanca	0.536317464	0.002511832
(b) (c)	ENSMUSG00000062270	Morf4l1b; Morf4l1	0.534632587	7.45E-05
(b) (c)	ENSMUSG00000025939	Ube2w	0.534620031	0.010912349
(b) (c)	ENSMUSG00000041974	Spidr	0.528372085	0.031073854
(b) (c)	ENSMUSG00000002221	Paxip1	0.524784379	0.00219332
(b) (c)	ENSMUSG00000027342	Pcna	0.510190225	0.000149795
(b) (c)	ENSMUSG00000036781	Rps27l	-0.511149122	0.000396513
(b) (c)	ENSMUSG00000055932	Fto	-0.542207412	0.000836506
(b) (c)	ENSMUSG00000025218	Poll	-0.55673431	0.010802132
(b) (c)	ENSMUSG00000051768	Xrcc1	-0.565059274	0.011650519
(b) (c)	ENSMUSG00000030750	Nsmce1	-0.568863795	0.028505041
(b) (c)	ENSMUSG00000030271	Ogg1	-0.583072498	0.005044913
(b) (c)	ENSMUSG00000019470	Xab2	-0.58735495	0.013516264
(b) (c)	ENSMUSG00000024735	Prpf19	-0.603078439	0.009525702
(b) (c)	ENSMUSG00000055401	Fbxo6	-0.605461249	0.034675231
(b) (c)	ENSMUSG00000002963	Pnkp	-0.608418421	0.009318974
(b) (c)	ENSMUSG00000024767	Otub1	-0.613242445	0.00175532
(b) (c)	ENSMUSG00000001524	Gtf2h4	-0.619546887	0.009397437
(b) (c)	ENSMUSG00000024740	Ddb1	-0.638154724	0.000264687
(b) (c)	ENSMUSG00000039615	Stub1	-0.651523074	0.012414939
(b) (c)	ENSMUSG00000040174	Alkbh3	-0.657991348	0.000263542
(b) (c)	ENSMUSG00000022400	Rbx1; Rbx1-ps	-0.681777796	0.007300222
(b) (c)	ENSMUSG00000003549	Ercc1	-0.70503675	0.000830829
(b) (c)	ENSMUSG00000047617	Paxx	-0.739402066	0.025341143
(b) (c)	ENSMUSG00000025374	Nabp2	-0.740551441	0.00038442
(b) (c)	ENSMUSG00000024906	Mus81	-0.740843757	0.000880794
(b) (c)	ENSMUSG00000051238	Swsap1	-0.745232976	0.000997283
(b) (c)	ENSMUSG00000006335	Tfpt	-0.769145049	0.002356025
(b) (c)	ENSMUSG00000073684	Faap20	-0.784304149	0.000270614
(b) (c)	ENSMUSG00000026162	-	-0.793055183	0.006926719
(b) (c)	ENSMUSG00000030034	Ino80b	-0.831592129	0.001189751
(b) (c)	ENSMUSG00000044339	Alkbh2	-0.833346258	0.011370675
(a) (b) (c)	ENSMUSG00000031095	Cul4b	0.938901225	3.79E-07
(a) (b) (c)	ENSMUSG00000027353	Mcm8	0.910799731	0.012568127
(a) (b) (c)	ENSMUSG00000031347	Cetn2	0.899437188	5.38E-06
(a) (b) (c)	ENSMUSG00000034023	Fancd2	0.885846108	0.000378505
(a) (b) (c)	ENSMUSG00000021635	Rad17	0.839230895	0.000202922
(a) (b) (c)	ENSMUSG00000025077	Dclre1a	0.826334362	0.004672021
(a) (b) (c)	ENSMUSG00000022314	Rad21	0.789659282	1.05E-10
(a) (b) (c)	ENSMUSG00000061607	Mdc1	0.714674087	0.00017233
(a) (b) (c)	ENSMUSG00000030983	Bccip	0.678298203	3.05E-05
(a) (b) (c)	ENSMUSG00000024151	Msh2	0.670732415	0.000316942
(a) (b) (c)	ENSMUSG00000001228	Uhrf1	0.63159937	2.11E-06
(a) (b) (c)	ENSMUSG00000039187	Fanci	0.58290451	0.042490722
(a) (b) (c)	ENSMUSG00000046591	Ticrr	0.570781198	0.004776598
(a) (b) (c)	ENSMUSG00000025358	Cdk2	0.513525522	0.003009294
(a) (b) (c)	ENSMUSG00000020415	Pttg1	-0.744583549	0.002444325
(a) (b) (c)	ENSMUSG00000031820	Babam1	-0.75298776	0.005483965
(a) (b) (c)	ENSMUSG00000025144	Cenpx	-0.789552562	0.003274342
(a) (b) (c)	ENSMUSG00000030744	Rps3	-0.829852846	0.000231516

Supplementary Table 4. Sequences of primers used in the study.

Primer pair	Name	Position	Sequence	Use
1	qGAPDH_m_01_F	F	CATCTTCCAGGAGCGAGACC	RTqPCR
	qGAPDH_m_01_R	R	CCTTCAAGTGGGCCCCG	RTqPCR
2	qP21_m_01_F	F	GCAGACCAGCCTGACAGATTT	RTqPCR
	qP21_m_01_R	R	CTGACCCACAGCAGAAGAGG	RTqPCR
3	qMyo1Cm_01_F	F	AAGCTCGCTGACCAGAAGAC	RTqPCR
	qMyo1Cm_01_R	R	AGCTGCACATGGTCTCCTTC	RTqPCR
4	p21 prom_m_A_F	F	TTCTGAAGAGGGGAGAGGGG	ChIPqPCR
	p21 prom_m_A_R	R	GTCCAGTTCTCAGACCACGG	ChIPqPCR
5	p21 prom_m_B_F	F	TATAAGGAGGCAGCTCGACG	ChIPqPCR
	p21 prom_m_B_R	R	GATCTGCGCCTGACTCCAAT	ChIPqPCR
6	p21 prom_m_C_F	F	AGAGGGAGCCTGAAGACTGT	ChIPqPCR
	p21 prom_m_C_R	R	CAGGATTGGACATGGTGCCT	ChIPqPCR
