# Supplementary material for: Nuclear myosin 1 activates p21 gene transcription in response to DNA damage through a chromatin-based mechanism
Source: Commun Biol. 2020 Mar 11;3:115. doi: 10.1038/s42003-020-0836-1 (PMC7066169; doi:10.1038/s42003-020-0836-1)
Supplement: Supplementary file 2 — Description of Additional Supplementary Files [file 42003_2020_836_MOESM2_ESM.pdf]

Description of additional supplementary items

File Name: Supplementary data 1

Description: Data source for all graphs in the paper
