# Supplementary material for: Nuclear myosin 1 activates p21 gene transcription in response to DNA damage through a chromatin-based mechanism
Source: Commun Biol. 2020 Mar 11;3:115. doi: 10.1038/s42003-020-0836-1 (PMC7066169; doi:10.1038/s42003-020-0836-1)
Supplement: Supplementary file 5 — Reporting Summary [file 42003_2020_836_MOESM5_ESM.pdf]

nature research

Corresponding author(s): Piergiorgio Percipalle

Last updated by author(s): Aug 21, 2019

Reporting Summary

Nature Research wishes to improve the reproducibility of the work that we publish. This form provides structure for consistency and transparency in reporting. For further information on Nature Research policies, see Authors & Referees and the Editorial Policy Checklist.

Statistics

For all statistical analyses, confirm that the following items are present in the figure legend, table legend, main text, or Methods section.

n/a	Confirmed	
☐	☒	The exact sample size (n) for each experimental group/condition, given as a discrete number and unit of measurement
☐	☒	A statement on whether measurements were taken from distinct samples or whether the same sample was measured repeatedly
☐	☒	The statistical test(s) used AND whether they are one- or two-sided<br>*Only common tests should be described solely by name; describe more complex techniques in the Methods section.*
☐	☒	A description of all covariates tested
☒	☐	A description of any assumptions or corrections, such as tests of normality and adjustment for multiple comparisons
☐	☒	A full description of the statistical parameters including central tendency (e.g. means) or other basic estimates (e.g. regression coefficient) AND variation (e.g. standard deviation) or associated estimates of uncertainty (e.g. confidence intervals)
☐	☒	For null hypothesis testing, the test statistic (e.g. F, t, r) with confidence intervals, effect sizes, degrees of freedom and P value noted<br>*Give P values as exact values whenever suitable.*
☒	☐	For Bayesian analysis, information on the choice of priors and Markov chain Monte Carlo settings
☒	☐	For hierarchical and complex designs, identification of the appropriate level for tests and full reporting of outcomes
☒	☐	Estimates of effect sizes (e.g. Cohen's d, Pearson's r), indicating how they were calculated

Our web collection on statistics for biologists contains articles on many of the points above.

Software and code

Policy information about availability of computer code

Data collection	n/a
Data analysis	RNA seq analysis, including quality trimming, was executed using the BioSAILs workflow execution system. The raw reads were quality trimmed using Trimmomatic (version 0.36) to trim low quality bases, systematic base calling errors, as well sequencing adapter contamination. FastQC was used to assess the quality of the sequenced reads pre/post quality trimming. Only the reads that passed quality trimming in pairs were retained for downstream analysis. The quality trimmed RNAseq reads were aligned to the Mus Musculus GRCm38 (mm10) genome using HISAT2 (version 2.0.4). The resulting SAM alignment files for each sequenced sample were then converted to BAM format and sorted by coordinate using SAMtools (version 0.1.19). The BAM alignment files were processed using HTseq-count using the reference annotation file to produce raw counts for each sample. The raw counts were then analyzed using the online analysis portal NASQAR (http://nasqar.abudhabi.nyu.edu/) in order to merge, normalize and identify differentially expressed genes. Differentially expressed genes by at least 2-fold (log2(FC)≥1 and adjusted p-value of <0.05 for upregulated genes and log2(FC)≤-1 and adjusted p-value of <0.05 for downregulated genes) between the NM1 WT and KO MEFs were subjected to Gene Ontology (GO) enrichment using DAVID Bioinformatics (https://david.ncifcrf.gov/). Venn diagram was produced by Bioinformatics & Evolutionary Genomics platform (http://bioinformatics.psb.ugent.be/webtools/Venn/).

For manuscripts utilizing custom algorithms or software that are central to the research but not yet described in published literature, software must be made available to editors/reviewers. We strongly encourage code deposition in a community repository (e.g. GitHub). See the Nature Research guidelines for submitting code & software for further information.

Data

Policy information about availability of data

All manuscripts must include a data availability statement. This statement should provide the following information, where applicable:

- Accession codes, unique identifiers, or web links for publicly available datasets
- A list of figures that have associated raw data
- A description of any restrictions on data availability

RNA-seq data on NM1 KO and WT primary mouse embryonic fibroblasts were deposited in the Gene Expression Omnibus (GEO) database under accession number GSE13350.

Field-specific reporting

Please select the one below that is the best fit for your research. If you are not sure, read the appropriate sections before making your selection.

☒ Life sciences ☐ Behavioural & social sciences ☐ Ecological, evolutionary & environmental sciences

For a reference copy of the document with all sections, see nature.com/documents/nr-reporting-summary-flat.pdf

Life sciences study design

All studies must disclose on these points even when the disclosure is negative.

Sample size	for RNA seq analysis 3WT and 3KO mouse embryonic fibroblasts were used. For each MEF cell line, 3 embryos of given genotype were harvested
Data exclusions	no data were excluded
Replication	all experiments in the study done at least in triplicates
Randomization	not relevant in our study
Blinding	RNA seq - library preparation and initial data analysis was done in blind manner<br>High-content screening - data analyzed in blind manner

Reporting for specific materials, systems and methods

We require information from authors about some types of materials, experimental systems and methods used in many studies. Here, indicate whether each material, system or method listed is relevant to your study. If you are not sure if a list item applies to your research, read the appropriate section before selecting a response.

Materials & experimental systems

n/a	Involved in the study	
☐	☒	Antibodies
☐	☒	Eukaryotic cell lines
☒	☐	Palaeontology
☒	☐	Animals and other organisms
☒	☐	Human research participants
☒	☐	Clinical data

Methods

n/a	Involved in the study	
☒	☐	ChIP-seq
☒	☐	Flow cytometry
☒	☐	MRI-based neuroimaging

Antibodies

Antibodies used	H3K9me3 (ab8898), H3K27ac (ab4729), H3K4me1 (ab8895), H3K4me3 (ab8580), H3K9ac (ab10812), γH2AX (ab2893), p53-K370 (ab183544), PCAF (ab12188), Set1 (ab70378), GAPDH (ab8245), nonspecific rabbit IgG isotype control (ab37415), Alexa Fluor 555 Goat Anti-Rabbit (ab150078) and Alexa Fluor 488 Goat Anti-Mouse (ab150117), HRP-fused Goat Anti-Rabbit (ab6721) and Rabbit Anti-Mouse (ab6728) were purchased from Abcam (Cambridge, MA, USA). The beta-tubulin antibody (sc-9104) was purchased from Santa Cruz (Dallas, Texas, USA). Antibody against NM1 has been previously characterized.
Validation	All commercial antibodies were tested, validated and used in several studies. Antibody against NM1 has been previously characterized and used in several papers (as for example: Almuzzaini, B., Sarshad, A. A., Farrants, A. K. & Percipalle, P. Nuclear myosin 1 contributes to a chromatin landscape compatible with RNA polymerase II transcription activation. BMC Biol 13, 35, doi:10.1186/s12915-015-0147-z (2015)

Eukaryotic cell lines

Policy information about cell lines

Cell line source(s)	Primary mouse embryonic fibroblasts derived from 13.5 day embryos from NM1 KO and WT mice (Venit et al. 2013). Immortalized mouse embryonic fibroblasts were purchased from ATCC (ATCC® CRL-2752).
Authentication	n/a
Mycoplasma contamination	tested-negative
Commonly misidentified lines (See ICLAC register)	n/a
